# Transcriptional Regulation of the Human IL5RA Gene through Alternative Promoter Usage during Eosinophil Development

**DOI:** 10.3390/ijms221910245

**Published:** 2021-09-23

**Authors:** Kimberly G. Laffey, Jian Du, Adam G. Schrum, Steven J. Ackerman

**Affiliations:** 1 Department of Biochemistry and Molecular Genetics (M/C 669), College of Medicine, University of Illinois at Chicago, 2074 Molecular Biology Research Building, 900 S. Ashland Ave., Chicago, IL 60607, USA; laffeyk@health.missouri.edu (K.G.L.); jiandu82@gmail.com (J.D.); 2 Department of Molecular Microbiology and Immunology, School of Medicine, University of Missouri, Columbia, MO 65212, USA; schruma@health.missouri.edu; 3 Department of Surgery, School of Medicine, University of Missouri, Columbia, MO 65212, USA; 4 Department of Biomedical, Biological, and Chemical Engineering, College of Engineering, University of Missouri, Columbia, MO 65211, USA

**Keywords:** eosinophil development, IL-5RA expression, alternative promoters

## Abstract

Regulation of the IL-5 receptor alpha (*IL5RA*) gene is complicated, with two known promoters (P1 and P2) driving transcription, and two known isoforms (transmembrane and soluble) dichotomously affecting the signaling potential of the protein products. Here, we sought to determine the patterns of P1 and P2 promoter usage and transcription factor occupancy during primary human eosinophil development from CD34^+^ hematopoietic stem cell progenitors. We found that during eosinophilopoiesis, both promoters were active but subject to distinct temporal regulation, coincident with combinatorial interactions of transcription factors, including GATA-1, PU.1, and C/EBP family members. P1 displayed a relatively constant level of activity throughout eosinophil development, while P2 activity peaked early and waned thereafter. The soluble IL-5Rα mRNA peaked early and showed the greatest magnitude fold-induction, while the signaling-competent transmembrane isoform peaked moderately. Two human eosinophilic cell lines whose relative use of P1 and P2 were similar to eosinophils differentiated in culture were used to functionally test putative transcription factor binding sites. Transcription factor occupancy was then validated in primary cultures by ChIP. We conclude that IL-5-dependent generation of eosinophils from CD34^+^ precursors involves complex and dynamic activity including both promoters, several interacting transcription factors, and both signaling and antagonistic protein products.

## 1. Introduction

The high-affinity interleukin (IL)-5 receptor is a heterodimeric protein consisting of an α subunit that binds specifically to the ligand, IL-5, and a β common (βc) subunit that is shared with the receptors for IL-3 and granulocyte-macrophage colony stimulating factor (GM-CSF) [1,2]. IL-5 signaling through its receptor is a critical event required for the differentiation, proliferation, recruitment and activation of the eosinophil [3,4]. The IL-5/IL-5R axis is further involved in pathologic eosinophilia. Excessive accumulation of eosinophils, both in tissues and peripheral blood, is a hallmark feature of allergic diseases, such as eosinophilic asthma and atopic dermatitis [5,6,7,8], and has been implicated in increasing the morbidity of such diseases [9]. Recognizing IL-5 signaling to be a critical event, humanized anti-IL-5 monoclonal antibodies (mepolizumab and reslizumab) have been developed and demonstrated clinical efficacy [10,11].

The IL-5Rα subunit exists in two isoforms: transmembrane and soluble [12,13]. The transmembrane isoform with its cytoplasmic tail is required for signal transduction from the heterodimeric IL-5R complex [3,4], whereas the soluble isoform is thought to bind and sequester IL-5 to inhibit signaling [1,14]. Being the IL-5-binding subunit in the surface receptor [15,16], transmembrane IL-5Rα represents the rate-limiting component of the IL-5 signaling pathway, and its regulation may constitute an important aspect of primary eosinophil development. 

Expression of IL-5Rα can be influenced by proinflammatory cytokines, including IL-3, IL-5, IL-9 and GM-CSF [17,18,19,20,21]. Regulation of IL-5Rα levels by these cytokines appears to occur, at least in part, at the transcriptional level [17]. Interestingly, the IL-5Rα level responds differently to exposure to these cytokines depending on maturation state along the eosinophilopoietic pathway: in early CD34^+^ eosinophil progenitors (EoP), IL-5 and IL-9 upregulate IL-5Rα mRNA and surface protein expression [18,20], while in mature peripheral blood eosinophils, IL-3, IL-5, and GM-CSF downregulate IL-5Rα mRNA and surface protein expression [17,19]. With two isoforms, and heterogeneous cellular responses, both healthy and pathologic, IL-5R regulation appears to be complex and remains incompletely understood.

IL-5-dependent eosinophil development requires transcription factors GATA-1, PU.1, and members of the C/EBP family, all essential for the commitment and terminal differentiation of myeloid progenitors to the eosinophil lineage [22,23,24,25,26,27]. Consistent with these observations, our group and others previously identified two functional promoters for the human IL5RA gene, P1 [28] and P2 [29], which we hypothesize may explain the connection between these transcription factors, IL-5Rα expression, and eosinophilopoiesis. According to in silico analyses, the P1 promoter appears to be a classical TATA-box containing promoter and has putative binding sites for GATA and C/EBP family members. Functionally, P1 activity has been shown to be potentially mediated by binding at an AP-1 site located at −440 to −432 [30]. Electrophoretic mobility shift assays (EMSA) demonstrated the AP-1 site to be in complex with cJun, CREB, and CREM. In addition, the binding of RFX proteins at a cis-regulatory element located at −430 and −421 is also essential for P1 activity [31]. In contrast, the P2 promoter lacks a TATA box, but is predicted to contain putative PU.1 and C/EBP family member binding sites. A unique 6 bp element located at −19 to −14 has been shown to be required for P2 activity in HL-60-C15 eosinophilic cells [29], although it is not yet known which transcription factor(s) bind(s) there. 

Still unknown are the temporal and combinatorial patterns by which transcription factors may interact with P1 and P2 promoters to effect IL-5Rα transcription during eosinophil differentiation. While both promoters have been shown to be active in some eosinophilic cell lines, the extent to which they might play specific or differential roles is unclear. Zhang et al. showed preferential utilization of the P2 promoter in butyrate-induced eosinophilic HL-60 cells [29]. This is consistent with the attractive hypothesis that P2 may be the preferred promoter post-eosinophil lineage commitment, and that P2 may also influence alternative splicing towards the transmembrane signaling-active transcript variant important for eosinophil differentiation. 

Here, we sought to determine the patterns of P1 and P2 promoter usage and transcription factor occupancy during primary human eosinophil development from CD34^+^ hematopoietic stem cell progenitors. The simplest initial hypotheses included the preferential expression of P2-driven transcripts and preferential expression of the transmembrane variant to promote IL-5R signaling, but the data showed that neither of these principles could fundamentally represent the time course of eosinophilopoiesis. P1 and P2 promoters of the *IL5RA* gene were both active and subject to distinct temporal regulation, coincident with combinatorial interactions of transcription factors, including GATA-1, PU.1, and C/EBP family members. It was the soluble transcript isoform of *IL5RA* that peaked earliest and showed the greatest magnitude fold-induction, while the signaling competent transmembrane transcript isoform peaked moderately. Two related human eosinophilic cell lines were identified whose relative use of P1 and P2 were similar to early- or late-stage eosinophils differentiated from CD34^+^ progenitors in culture, and these lines were used to identify and functionally test promoter sequences and transcription factor associations, whose presence in primary cultures was subsequently validated by ChIP assays. The model that emerges to connect *IL5RA* transcription with eosinophilopoiesis involves complex and dynamic activity, including both P1 and P2 promoters, several interacting transcription factors, and both signaling and antagonistic protein products.

## 2. Results

### 2.1. Structure of the Human IL5RA Genetic Locus and Its Transcripts

**Figure 1:**
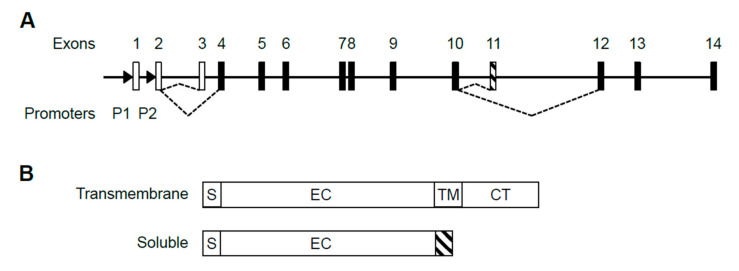
Structure of the human *IL5RA* gene, its alternative transcripts and protein isoforms. (**A**) Alternative splicing choice resulting in the soluble or transmembrane splice variant is indicated by dashed lines. Open boxes represent untranslated exons and black boxes represent translated exons. Exon 11 (striped) is soluble-specific while exons 12–14 are transmembrane-specific. Promoters P1 and P2 are located upstream of exons 1 and 2 as indicated by arrowheads. (**B**) IL-5Rα exists in two protein isoforms. The soluble isoform lacks transmembrane and cytoplasmic domains and instead has a soluble isoform-specific domain (striped) encoded by exon 11. **S**: signal peptide. **EC**: extracellular domain; **TM**: transmembrane domain; **CT**: cytoplasmic tail.

The human *IL5RA* gene is comprised of 14 exons, with the first 3 exons being non-coding (Figure 1A). Transcription of the gene is driven by two promoters, P1 and P2, producing at least 5 alternatively spliced transcripts (Gene ID: 3568) which are translated into two protein isoforms, either soluble or transmembrane [20] (Figure 1B). Upon sequencing the 5′ ends, we confirmed published 5′ sequences of P1-derived and P2-derived transcripts, having distinct 5′ UTRs with only P1-derived transcripts containing exon 1 (Figure S1) [28,29]. To date, the presence of exon 1 represents the only detectable difference between P1-derived and P2-derived transcripts. No P2-specific exon has been discovered in the transcripts. 

### 2.2. Differential Usage of P1 and P2 Promoters

**Figure 2:**
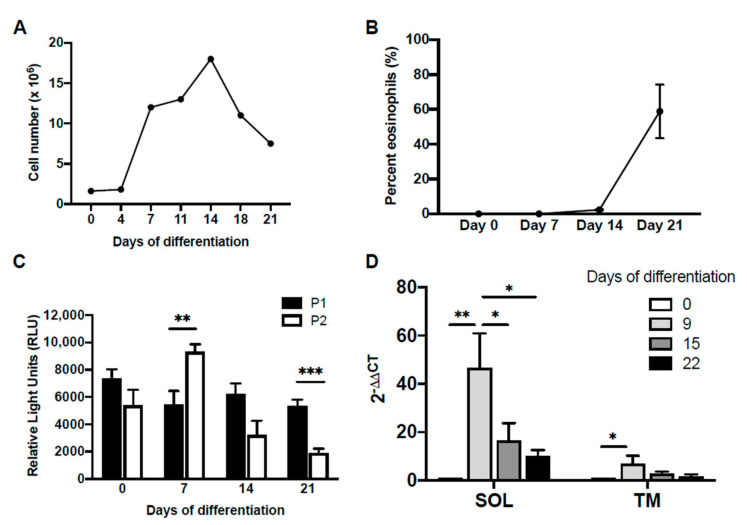
Differential promoter activity during IL-5-dependent differentiation of primary eosinophils from human cord blood-derived CD34^+^ cells. CD34^+^ progenitors were first cultured in suspension in SCF, FLT-3, TPO, and GM-CSF for 4 days, followed by only IL-3 and IL-5 to induce differentiation to the eosinophil lineage. (**A**) Cell proliferation and (**B**) percentage of mature eosinophils in culture determined on differential counts using Fast Green/Neutral Red staining is shown. (**C**) Changes in differential promoter activity during eosinophil differentiation were determined by transiently transfecting differentiating cells on days 0, 7, 14, and 21, with IL-5RαP1 or IL-5RαP2 luciferase promoter reporter constructs with a pRL-CMV expression vector as an internal control for transfection efficiency. Promoter activities were measured 6 h post-transfection. Raw measurements in relative light units were first normalized by the dual luciferase method followed by subtraction of background luciferase activity (reading from promoterless vector control). Data are shown as mean relative light units above background for 2–4 independent experiments. SD, standard deviation. (**D**) Quantitative real-time PCR was performed to measure mRNA levels of the IL-5Rα soluble and transmembrane splice variants during differentiation. Data are shown as fold changes in mRNA levels over day 0 for one representative experiment. SD, standard deviation. * *p* < 0.05 ** *p* < 0.01 *** *p* < 0.001.

It has previously been shown that P2 promoter activity was exclusive to eosinophilic HL-60-C15 cells [29]. We therefore hypothesized that *IL5RA* promoter usage in the developing eosinophil would undergo a switch from P1 to P2, with progressive differentiation. To determine if there is temporal regulation of preferential promoter usage, we performed promoter–reporter studies using CD34^+^ hematopoietic stem cells undergoing IL-5-dependent eosinophilopoiesis (Figure 2A,B). In this system, the differentiation age of the culture could be determined by assessing the presence of mature eosinophils obtained under the influence of IL-5 (Figure 2B). When promoter activities were measured in differentiating eosinophil progenitors in this system (Figure 2C), P1 was observed to be active throughout differentiation. In contrast, P2 exhibited a transient increase in activity to surpass that of P1 on day 7 before getting rapidly attenuated as differentiation continued and completed by day 21. Both soluble and transmembrane *IL5RA* transcription underwent a dramatic increase early during differentiation, before decreasing to more basal levels (Figure 2D).

**Figure 3:**
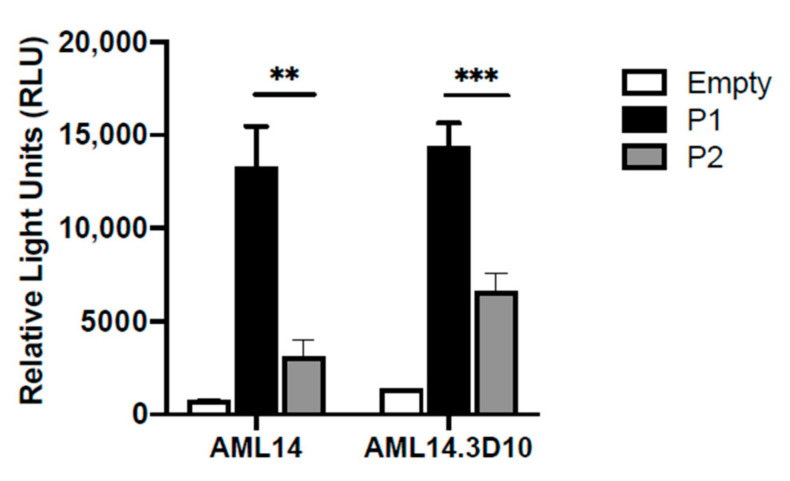
Differential *IL-5RA* P1 and P2 promoter activities in two eosinophilic cell lines. P1 and P2 are both constitutively active in the eosinophilic myeloblastic line AML14 and in the eosinophilic myelocytic line AML14.3D10. ** *p* < 0.01 *** *p* < 0.001.

Furthermore, we observed a similar predominance of P1 promoter activity in 2 eosinophilic cell lines: the less differentiated myeloblastic cell line AML14; and the more differentiated myelocytic cell line AML14.3D10 [32] (Figure 3). This confirmed the validity of using the AML14 system as a model to further characterize *IL5RA* promoter regulation during eosinophilopoiesis.

### 2.3. Promoter Regulation by Differential Occupancies of Transcription Factors

**Figure 4:**
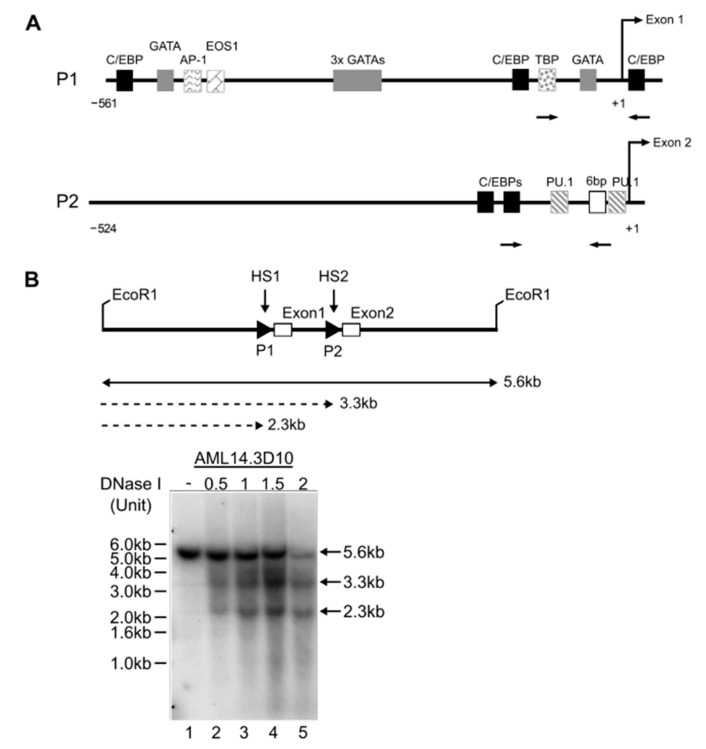
Cis regulation of the *IL5RA* P1 and P2 promoters. (**A**) In silico prediction of transcription factor binding sites and consensus sequences specific to the functionally active region of the in P1 and P2 region promoters. The transcription start site is designated +1. Arrows represent positions of primers used for ChIP analyses. (**B**) Identification of two DNase I hypersensitive sites HS1 and HS2 in AML14.3D10. HS1 and HS2 correspond to positions of P1 and P2 as indicated by DNA fragments of 2.3 kb and 3.3 kb, respectively.

In silico analyses revealed consensus binding sites for transcription factors implicated in eosinophil development in the P1 and P2 promoters of *IL5RA* (Figure 4A). A pre-requisite for transcriptional control through transcription factor occupancy is an open chromatin structure allowing access to chromatin. Consistent with both P1 and P2 promoters being active in AML14.3D10 eosinophils, two DNAse I hypersensitive sites corresponding to the positions of P1 and P2 were found (Figure 4B). We subsequently validated some of these predicted sites by mutagenesis studies and electrophoretic mobility shift assays (EMSAs), as described below. 

#### 2.3.1. GATA-1 Binding Sites

**Figure 5:**
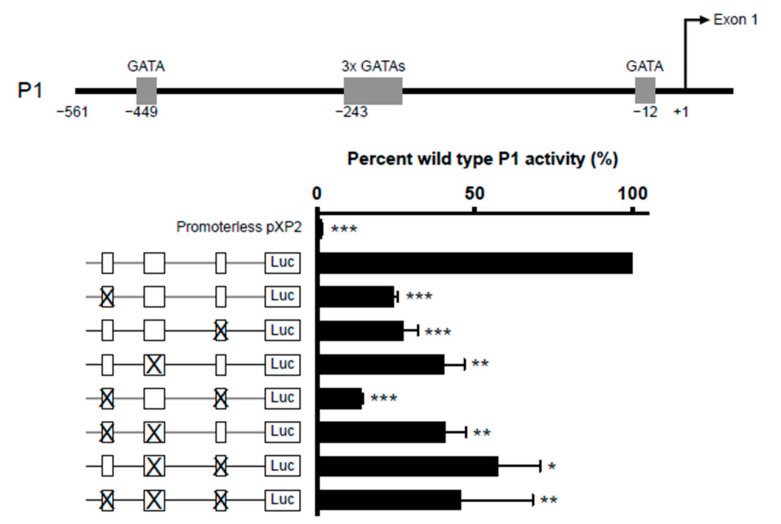
Combinatorial mutagenesis reveals positive regulation of *IL5RA* P1 activity by multiple GATA sites. Substitution mutations were introduced into the GATA sites in the *IL5RA* P1 promoter at positions −449, −243 (triple GATA site) and −12 singly or in combination. For the mutation of the triple GATA site, all three GATA sites were mutated. AML14.3D10 eosinophilic myelocytes were transiently transfected with the IL-5RαP1 luciferase promoter reporter construct containing the wild-type or mutated GATA sites with a pRL-TK expression vector as an internal control for transfection efficiency. Promoter activities were measured 6 h post-transfection and were normalized by the dual luciferase method. Data are shown as mean percent wild-type P1 activity for three independent experiments. * *p* < 0.05 ** *p* < 0.01 *** *p* < 0.001.

Five GATA-1 sites were predicted in the *IL5RA* P1 promoter. They are comprised of two single GATA sites situated at positions −449 and −12 and a cluster of three closely situated GATA sites starting at position −243. (Figure 5, upper panel). To determine which of these sites are functionally important, we created IL-5RαP1 promoter reporter constructs containing single or multiple GATA site mutations and assessed their ability in activating transcription in the eosinophilic cell line AML14.3D10 (Figure 5, lower panel). All GATA sites were seen to contribute to P1 activity, as when each site was mutated singly or in combination, promoter activity was significantly decreased relative to wild-type promoter.

#### 2.3.2. C/EBP Binding Site

**Figure 6:**
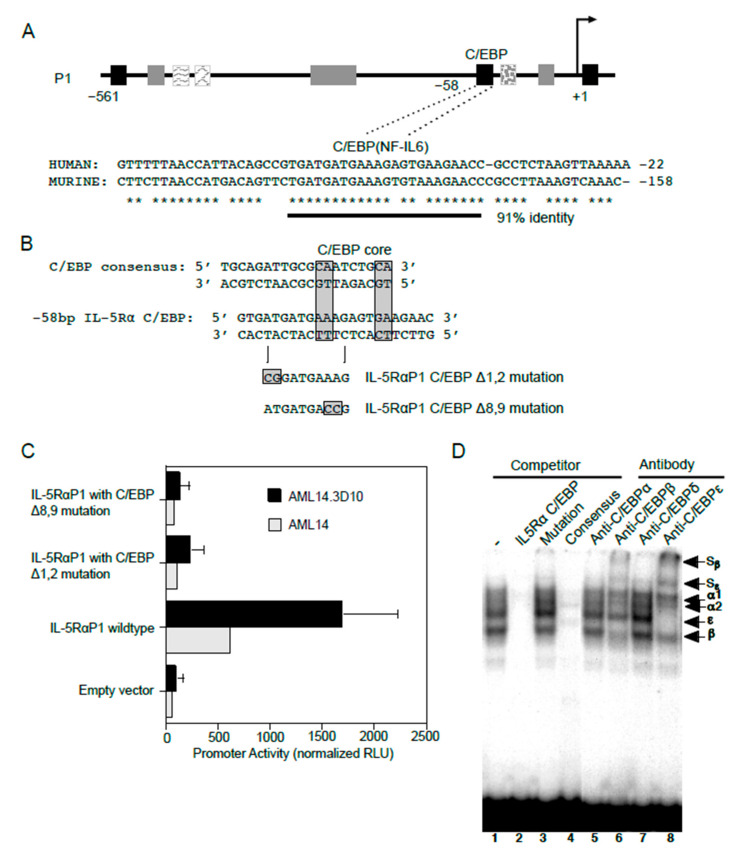
C/EBP functionally binds and regulates *IL5RA* P1 promoter activity in eosinophilic cell lines. (**A**) Sequence alignment of the human *IL5RA* P1 and putative murine *IL5RA* promoters highlights the conserved C/EBP-binding site in both promoters in a region of 91% sequence identity. Conserved bases are indicated by asterisks. (**B**) Alignments of the *IL5RA* P1 and consensus C/EBP binding sites, and mutations generated in the C/EBP site of the IL-5RαP1-pXP2 promoter construct. (**C**) AML14 eosinophilic myeloblasts and AML14.3D10 eosinophilic myelocytes were transiently transfected with the IL-5RαP1 luciferase promoter reporter construct, containing either the wild-type or mutated C/EBP-binding site, along with a pRL-TK expression vector as an internal control for transfection efficiency. Promoter activities were measured 6 h post-transfection and were normalized by the dual luciferase method. Data are shown as mean (±SD) relative light units for three independent experiments. (**D**) AML14.3D10 nuclear extract (3 μg) was used to demonstrate specificity of various C/EBP protein-DNA complexes. Complex formation was specifically inhibited by a 50-fold molar excess of unlabeled IL-5RαP1 C/EBP (lane 2) and consensus C/EBP site probes (lane 4), but not by a C/EBP site mutation (lane 3). Complex formation was strongly inhibited by antibodies to C/EBPβ and C/EBPε with visible supershifted complexes (S_β_ and S_ε_, lanes 6 and 8), but not by antibodies to C/EBPα or C/EBPδ (lanes 5 and 7). Specific proteins in protein-DNA complexes are indicated.

In addition to GATA-1 sites, consensus C/EBP binding sites were also predicted in the *IL5RA* P1 promoter. Among these, one C/EBP site situated at −58 bp of the P1 promoter is conserved between the human and putative murine promoter sequences (Figure 6A), providing greater confidence for the presence of a functional binding site at this location. To test if C/EBP functionally binds at this location, two promoter reporter gene constructs each containing a specific two-nucleotide mutation were generated in this putative C/EBP binding site using site-directed mutagenesis (Figure 6B). When analyzed by transfection into AML14 and AML14.3D10 cells, both mutations (Δ1,2 and Δ8,9) completely abolished P1 promoter activity as compared to the WT promoter, indicating that this sequence is likely a functional C/EBP binding site (Figure 6C).

We next performed EMSAs for the endogenously expressed C/EBPs using nuclear extract from AML14.3D10 eosinophils (Figure 6D). In this cell line, greatest complex formation with the *IL5RA* P1 C/EBP site probe was observed for C/EBPβ and C/EBPε. This was confirmed by the presence of supershifted complexes when antibodies to C/EBPβ and C/EBPε were added. The protein-DNA complexes were not supershifted by antibodies to C/EBPα or C/EBPδ. The C/EBPβ and C/EBPε antibody supershifts are also consistent with a likely presence of C/EBPβ/ε heterodimers, since antibodies to either of these C/EBPs reduced the intensity of complex formation for the other C/EBP family member.

### 2.4. Dynamic Occupancy of Transcription Factors during Eosinophil Differentiation

**Figure 7:**
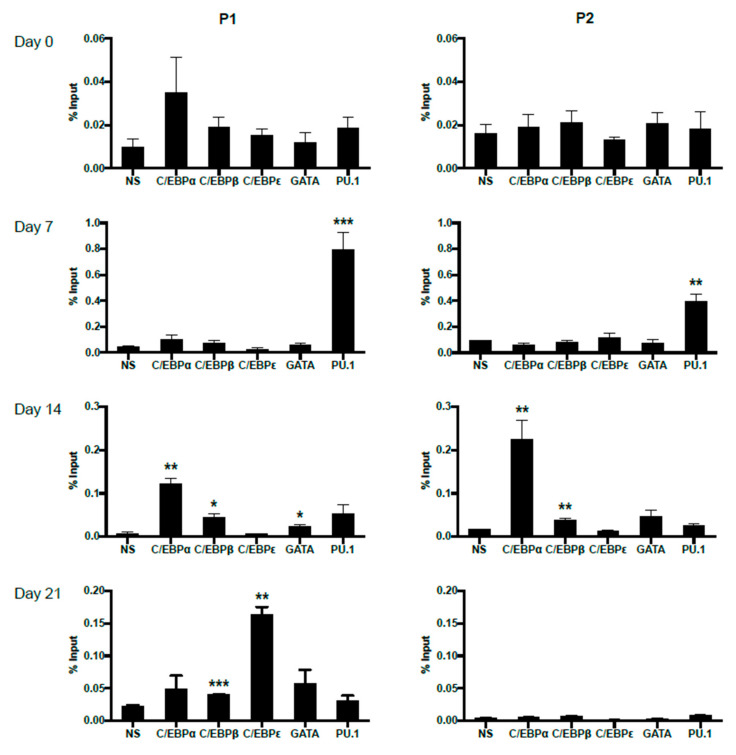
Differential in vivo occupancy of the *IL5RA* P1 and P2 promoters by important transcription factors in differentiating CD34^+^ progenitors as demonstrated by chromatin immunoprecipitation (ChIP). Chromatin immunoprecipitation was performed at days 0, 7, 14, and 21 of differentiating eosinophil progenitors to follow dynamic changes in transcription factor binding to the *IL5RA* P1 (left) and P2 (right) promoters. One representative experiment is shown out of 2–3 independent experiments. NS: non-specific Ig control. Significant enrichment by each antibody was compared to NS. * *p* < 0.05 ** *p* < 0.01 *** *p* < 0.001.

In order to trace the potentially changing occupancies of important transcription factors on P1 and P2 promoters during primary eosinophil differentiation from CD34^+^ hematopoietic stem cells, we performed ChIP analyses on eosinophil progenitors at days 0, 7, 14 and 21 of IL-5-induced differentiation (Figure 7). For both P1 and P2, no statistically significant binding was observed for the examined transcription factors at day 0, likely due to lack of IL-5Rα expression pre-commitment to the eosinophil lineage. As differentiation proceeded, P1 was observed to be bound first by PU.1 at day 7, then C/EBPα, C/EBPβ, and GATA-1 at day 14. On day 21, P1 was bound by C/EBPβ and C/EBPε. P2 was observed to be bound by PU.1 at day 7, then C/EBPα and C/EBPβ at day 14. No statistically significant occupancy of P2 was observed at day 21, consistent with the attenuation of P2 promoter activity late in eosinophil differentiation (Figure 2C).

## 3. Discussion

Differential promoter usage and alternative splicing represent an elegant regulatory mechanism to provide tissue- and/or developmental stage-specific gene expression and transcript diversity from a single genetic locus. Such context-dependent transcription is critical during development, and aberrant promoter usage has been implicated in various diseases (reviewed in [33]). Several genes important in eosinophil development are subject to gene expression modulation through alternative promoter usage. *CEPBE* (encodes C/EBPε) transcription is driven by 2 alternative promoters and through alternative splicing, yields protein isoforms with different functions in myeloid differentiation [26,34]. *IL1RL1* (encodes the IL-33 receptor ST2) similarly uses alternative promoters coupled to alternative splicing to affect cell type-specific gene expression [35,36]. In the present study, we show that the transcription of the human *IL5RA* gene is temporally regulated during eosinophil development through the differential usage of two alternative promoters.

It has been previously reported that the P2 promoter was active in eosinophilic HL-60-C15 cells and not in non-eosinophilic cell lines [29]. We hypothesized that P1 and P2 might represent “early” and “late” promoters, respectively, of eosinophil development. However, our data demonstrate that *IL5RA* alternative promoter usage is more complex. Using an ex vivo IL-5 induced eosinophil differentiation system, we show that the P1 promoter is active throughout the course of differentiation. The P2 promoter, by contrast, exhibits a transient increase in activity that exceeds P1 activity on day 7 of IL-5 induced eosinophil differentiation and is subsequently attenuated. In contrast to the cited butyrate-induced HL-60 differentiation model, we observed that in primary eosinophilopoiesis, the P1 promoter is a prominent promoter with stable usage. This observation also described both eosinophil-committed AML14 myeloblasts and eosinophil-differentiated AML14.3D10 myelocytes, consistent with the idea that these cell lines likely represent the early and late phases of eosinophil differentiation. These data emphasize that differential P1 and P2 promoter usage dynamically changes depending on stage-specific cellular maturation states.

The sustained activity of P1 may hint at its role as the “housekeeping” promoter that maintains IL-5Rα expression in both eosinophil progenitors and mature eosinophils. Interestingly, peak P2 activity coincides with a dramatic induction of *IL5RA* soluble transcripts. This suggests that P2 may be required to provide a “boost” for the developing eosinophil progenitor to bring about the marked increase in mRNA level. It has been suggested that the soluble IL-5Rα isoform acts to neutralize the effects of IL-5 on eosinophils and their progenitors, mitigating a potential over-exuberant eosinophilic response [14]. Such negative regulation of IL-5 signaling by increasing soluble IL-5Rα is suggested to come from the proteolytic cleavage of transmembrane IL-5Rα at the cell surface [37]. However, it can be speculated that in early eosinophil progenitors with very low transmembrane IL-5Rα expression, soluble IL-5Rα is not generated from proteolytic cleavage alone, but instead must come from increased transcription.

Mechanistically, we postulate that differential promoter usage for the *IL5RA* gene is mediated by the combinatorial actions of dynamically expressed transcription factors during eosinophil development. We experimentally validated a subset of predicted transcription factor binding sites in AML14.3D10 eosinophilic myelocytes, and used ChIP to follow the kinetics of transcription factor occupancy in differentiating CD34^+^ cells. Here, we observed a lack of occupancy on both promoters at day 0. This may be due to the immaturity and lack of eosinophil lineage commitment of the CD34^+^ cells at this point, only a small percentage of which represent IL-5Rα^+^ committed EoPs. The subsequent occupancy of P1 by PU.1, GATA-1, C/EBPα, C/EBPβ, and finally C/EBPε late in differentiation is in keeping with established roles of these transcription factors during human eosinophil development (reviewed in [38,39,40,41]). Similarly, occupancy of P2 begins at day 7 by PU.1. Unlike P1, P2 lacks a TATA-box, but is predicted to have PU.1 sites close to the transcription start site. PU.1 has been shown to be able to recruit the transcription machinery to the promoter of the toll-like receptor 4 (*TLR4*) gene to initiate transcription [42]. The *IL5RA* P2 promoter could potentially be another example of transcription initiation mediated by PU.1. Finally, the eventual absence of transcription factor occupancy of P2 on day 21 of differentiation reflects the attenuation of P2 activity observed.

Whereas we observed the temporal regulation of *IL5RA* promoter activity in IL-5-mediated eosinophil differentiation of CD34^+^ progenitors, we were not able to discern any clear influence of preferential promoter usage over alternative splicing in producing transmembrane vs. soluble transcript variants. This may be a limitation of the ex vivo culture system in recapitulating the full range of cellular states, tissue localization, and growth factor/nutrient milieu to which developing and mature eosinophils respond in vivo. Notwithstanding the limitations, we favor a model in which the data accurately reflect the possibility of changing differential promoter usage coupled to alternative splicing, as a result of changing cellular states. Indeed, the expression of IL-5Rα mRNA isoforms is altered in a number of eosinophilic disorders. In patients with nasal polyposis (NP), the ratio of soluble to transmembrane IL-5Rα mRNA in polyp tissue was greater in NP patients with asthma than in patients without asthma [43]. Furthermore, the level of soluble transcript variant in polyp tissue was positively correlated with tissue eosinophilia, while the level of the transmembrane variant saw a negative correlation [43]. In a separate cohort of patients with varied eosinophilic disorders, the downregulation of the transmembrane transcript variant was also seen in blood eosinophils when compared to healthy controls [44]. It is unknown if the observed changes in the levels of different transcript variants is due to differential promoter usage when eosinophils are exposed to healthy vs. diseased microenvironments. Furthermore, the preferential promoter and splicing status in response to changing IL-5 levels are unknown during treatment. The inability of mepolizumab (anti-IL-5) to completely deplete tissue eosinophils [45,46,47] implies reduced IL-5 responsiveness in these eosinophils. It is further possible that these eosinophils may be of a different subtype than those affecting disease [48]. Therefore, elucidating alternative promoter usage and alternative splicing as a function of tissue location, disease state, and subtype of eosinophils could have important implications for treatment strategies.

## 4. Materials and Methods

### 4.1. Cell Culture

AML14 myeloblasts and AML14.3D10 eosinophilic myelocytes were maintained in RPMI 1640 supplemented with 8% fetal bovine serum, 2 mM L-glutamine, 1 mM sodium pyruvate and 50 μM β-mercaptoethanol without antibiotics. The cells were passed every 3–4 days and maintained at 0.3–1.0 × 10^6^ cells/mL in a humidified incubator with 5% CO_2_ at 37 °C.

### 4.2. Differentiation of CD34^+^ Progenitors

CD34^+^ cells purified from human umbilical cord blood (obtained from the New York Blood Center, New York, NY, USA) were cultured in Iscove’s modified Dulbecco medium (Lonza, Walkersville, MD, USA) supplemented with 10% fetal bovine serum, 2 mM L-glutamine, 50 μM β-mercaptoethanol, 10U/mL penicillin and 10 μg/mL streptomycin at 0.3 × 10^6^ cells/mL. To induce eosinophil differentiation, the cells were first expanded in SCF (50 ng/mL), Flt3-L (50 ng/mL), TPO (50 ng/mL), and GM-CSF (0.1 nM) for the first 4 days. Thereafter, cells were cultured in only IL-3 and IL-5 (0.1 nM each), with medium being replenished every 3–4 days. The differentiation stage of the culture was determined by assessing the percentage of mature eosinophils using differential counts of cultured cells stained with Fast Green/Neutral Red. All cytokines were purchased from R&D Systems (Minneapolis, MN, USA).

### 4.3. Reporter Constructs and Expression Vectors

The pXP2-IL-5RαP1 promoter construct containing bp −561 to +51 of the human IL-5Rα subunit P1 promoter has been previously described in detail [28,49]. Constructs containing two different mutations of the functional C/EBP site were generated by PCR mutagenesis and constructs containing single and multiple GATA site mutations were generated by site-directed mutagenesis (see below).

The pGL4.20 promoter constructs were generated by introducing restriction sites through the PCR amplification of existing promoter constructs and subcloning into the pGL4.20 vector (Promega, Madison, WI, USA). Specifically, the IL-5RαP1 promoter was amplified from the pXP2-IL-5RαP1 construct and subcloned using HindIII sites. The IL-5RαP2 promoter (bp −485 to +35) was amplified from a previously described construct [29] kindly provided by Dr. Ji Zhang (Schering-Plough Research Institute, Kenilworth, NJ, USA) and subcloned using XhoI and HindIII sites. All constructs were confirmed by sequencing.

### 4.4. Site-Directed Mutagenesis

#### 4.4.1. C/EBP Site Mutagenesis

Mutation of the C/EBP site in the *IL5RA* P1 promoter region was performed by oligonucleotide-directed PCR mutagenesis using the wild-type pXP2-IL-5RαP1 as a template (primers listed in Table 1). Primer 1 was used with either Primer 2 which contained the C/EBP Δ1,2 mutation or Primer 3 which contained the C/EBP Δ8,9 mutation to generate PCR fragment I. Primer 4 was used with either Primer 5 or Primer 6 which are the reverse complementary strands of Primer 2 and Primer 3 respectively, to generate PCR fragment II. Conditions for the first round PCR reaction were as follows: 1 min at 94 °C, 1.5 min at 50 °C and 2 min at 72 °C. Molar equivalents of PCR fragments I and II were annealed in a second round PCR for 2 cycles consisting of 1 min at 94 °C, 20 min at 61 °C and 10 min at 72 °C. The PCR product was then used as template in a third round PCR reaction performed using Primer 1 and Primer 6 for 30 cycles, consisting of 1 min at 94 °C, 1.5 min at 61 °C and 2min at 72 °C. The final PCR product was purified using a PCR purification kit (QIAGEN) and cloned into KpnI and XhoI sites of the pXP2 vector.

#### 4.4.2. GATA Site Mutagenesis

The 5 GATA sites in the wild type pXP2-IL-5RαP1 promoter construct were modified by oligonucleotide-directed mutagenesis using the QuikChange II mutagenesis kit (Agilent Technologies, Santa Clara, CA, USA) following manufacturer’s instructions. Multiple GATA site mutants were made through sequential mutagenesis, starting from a single GATA site mutant. All mutants were confirmed by sequencing in both directions and primers used are listed in Table 2.

### 4.5. Transient Transfections and Transactivations

All DNA plasmids for transfection were prepared by alkaline lysis maxi-preparation followed by CsCl_2_ purification. Transient transfections of the AML14 and AML14.3D10 cell lines were carried out at 1.5 × 10^7^cells/0.5ml in RPMI-1640 by electroporation at 280 V, 960 μF as previously optimized [28]. Then, 15 μg of experimental plasmid DNA was cotransfected with 0.5 μg pRL-TK or pRL-CMV Renilla luciferase control vector. For the transfection of differentiating CD34^+^ EoP, 1.0 × 10^6^ cells were co-transfected with 4.8 μg of experimental plasmid DNA and 0.2 μg of pRL-CMV control vector using the AMAXA nucleofection system according to the manufacturer’s protocol optimized for CD34+ cells (Program U-008, Lonza, Walkersville, MD, USA). Cell lysates were prepared 6 h post-transfection and promoter activities measured using the Dual Luciferase Assay system according to the manufacturer’s instructions (Promega, Madison, WI, USA). Dual luciferase activities (firefly and Renilla) were measured as relative light units (RLU) using an EG&G Berthold Lumat LB 1507 luminometer. Readings were normalized for transfection efficiency using Renilla luciferase activity.

### 4.6. Whole Cell Lysates and Nuclear Extracts

AML14.3D10 cells were lysed for one hour at 4 °C in a buffer containing 0.5–1% Triton X-100, 150 mM NaCl, 1 mM EDTA, 200 μm sodium orthovanadate, 50 mM HEPES, 10 mM sodium pyrophosphate, 100 mM sodium fluoride, 1.5 mM MgCl_2_, 10% glycerol, 1 mM phenylmethylsulfonyl fluoride (PMSF), 10 μg/mL aprotinin, and a Complete Mini protease inhibitor cocktail tablet (Roche, Germany), as previously described [50]. Whole cell lysates were collected by centrifugation at 14,000× *g* for 20 min at 4 °C to remove cell debris and lysates were stored at −80 °C. Nuclear extracts were prepared by the method of Dignam et al. with minor modifications, including the addition of protease inhibitor cocktail tablets (Roche, Germany), PMSF (0.5 mM) and diisopropylfluorophosphate (DFP) (1 mM) to both the resuspension and lysis buffers. The protein concentration of nuclear or whole cell extracts was determined by the BCA method (Pierce Thermo Scientific, Rockford, IL, USA).

### 4.7. EMSA

The double-stranded oligonucleotide probes for EMSAs included the *IL5RA* C/EBP site (5′-GTGATGATGAAAGAGTGAAGAAC-3′) and the G-CSFR consensus C/EBP site (5′-TGCAGATTGCGCAATCTGCA-3′). All oligonucleotides were synthesized and purified by Integrated DNA Technologies, Inc. (IDT, Coralville, IA, USA). To generate probes for EMSA, 10 pmol of the double-stranded oligonucleotide was end-labeled with α-^32^P-dATP (PerkinElmer NEN, Naperville, IL, USA) using T4 polynucleotide kinase, and the probes were purified on a 15% polyacrylamide gel as previously described [50]. For gel mobility shift assays, the DNA binding reactions were carried out at room temperature for 30 min in 20 μL final volume containing the labeled oligonucleotide probe (~10,000 cpm), 1 μL of nuclear extract (2 μg total protein), 2 μg of poly(dI⋅dC) in 20 mM HEPES pH7.9, 50 mM KCl, 2 mM MgCl_2_, 1 mM DTT, 0.5 mM EDTA, 5% glycerol. For competition experiments, an excess of unlabeled double-stranded oligonucleotide competitor (50-fold excess over the labeled oligonucleotide probe) was added prior to the hot probe and incubated for 10 min. For antibody supershift assays, 1 μg of antibody specific for C/EBPα, β, δ or ε (Santa Cruz Biotechnology Inc., Santa Cruz, CA, USA) was first incubated with the nuclear extracts in 10 μL of 10 mM Tris and 150 mM NaCl at room temperature for 1 h and then added to the binding reaction for the gel mobility shift assay. In all cases, the DNA probe was added last. The entire reaction mixture was loaded on a 4% polyacrylamide gel that had been pre-electrophoresed for 1 h. Electrophoresis was carried out at 11 V/cm in a 4 °C room. Gels were dried and subjected to autoradiography using a Molecular Dynamics PhosphorImager.

### 4.8. ChIP Analysis

ChIPs were performed using a modification of previously published methods for limited cell numbers [51,52]. Moreover, 1.0 × 10^5^ CD34^+^ cells at 0, 7, 14, and 21 days of eosinophil differentiation were used for each ChIP analysis. Nuclear chromatin and protein complexes were cross-linked for 10 min in 1% formaldehyde at room temperature; crosslinking was stopped by adding 125 mM glycine. The cells were then washed twice in ice-cold 1xPBS, resuspended in 200 μL lysis buffer (50 mM Tris-HCl, pH 8, 10 mM EDTA, 1% SDS) supplemented with protease inhibitor (Roche) and 1 mM PMSF and incubated on ice for 5 min. Chromatin was sheared to within 500bp fragments by sonication using a Branson 450 Sonifier outfitted with a microtip. The efficiency of sonication was checked on an agarose gel.

Antibody-paramagnetic bead complexes were prepared using Dynabeads Protein G (Invitrogen, Grand Island, NY, USA) following the manufacturer’s instructions. All antibodies used for immunoprecipitation were rabbit polyclonal antibodies purchased from Santa Cruz Biotechnology Inc. (Santa Cruz, CA, USA). For immunoprecipitation with each antibody, chromatin (10^5^ cell equivalents) was mixed with antibody-bead complexes and incubated for 2 h at 4 °C with rotation. After incubation, the bead complexes were captured against a strong magnet, washed four times with wash buffer (RIPA buffer, 5% BSA) and three times with TE buffer (10 mM Tris-HCl, pH 8.0, 10 mM EDTA).

The bead complexes were vortexed gently in 100 mM NaHCO_3_ for 15 min to extract the protein-DNA complexes. Matched input samples were also prepared in 100 mM NaHCO_3_. To reverse crosslinks, 250 mM NaCl was added to the eluted protein-DNA complexes and incubated at 95 °C for 15 min. Samples were digested with 200 μg/mL RNase A at 37 °C for 1h and subsequently with 50 μg/mL Proteinase K at 50 °C for 1 h. Finally, DNA was extracted using phenol:chloroform.

### 4.9. Quantitative Real-Time PCR

Quantitative real-time PCR was performed in triplicate on an iCycler iQ5 system (Biorad, Hercules, CA, USA) with EvaGreen Real-Time PCR kits (Feldan, QC, Canada). For the quantification of IL-5Rα transcripts, total RNA from 1.0 × 10^6^ CD34^+^ cells at each time point was isolated using Trizol (Invitrogen, Grand Island, NY, USA) according to manufacturer’s protocol. cDNA was synthesized from 250 ng of total RNA using RevertAid First Strand cDNA synthesis kit (Thermo Scientifc, Pittsburg, PA, USA). Results were normalized to the internal control β_2_M. Conditions and primers used have been described elsewhere [34]. For the analysis of immunoprecipitated DNA, the following conditions were used with primers listed in Table 3: 15 min at 95 °C followed by 40 cycles, consisting of 20 s at 95 °C, 40 s at 60.4 °C, 15 s at 72 °C. The β-actin promoter was amplified as a control for ChIP specificity [53].

### 4.10. In Silico Prediction of Transcription Factor Binding Sites

The prediction of putative transcription factor binding sites within the *IL5RA* P1 and P2 promoters was performed using the PROMO algorithm [54,55], which utilized the transcription factor database TRANSFAC version 8.3 [56].

### 4.11. Statistical Analysis

Unpaired Student’s t-test was used to compare two means and one-way ANOVA followed by the Tukey test was used to compare multiple means. Differences were considered statistically significant at *p* < 0.05. Unless otherwise stated, error bars represent SEM. Statistical analyses were done with Prism 8 (GraphPad Software, San Diego, CA, USA).

## Figures and Tables

**Table 1:** Primers used in C/EBP site mutagenesis. The positions of mutations are underlined.

	Primer Sequence	Mutations
Primer 1	5′-GGTACCAGACCTGCTCACAAAGC-3′	
Primer 2	5′-GTTCTTCACTCTTTCATCCGCAC-3′	Δ1,2
Primer 3	5′-GTTCTTCACTCGGTCATCATCAC-3′	Δ8,9
Primer 4	5′-CCGCTCGAGAAATGCGGTGGCCAT-3′	
Primer 5	5′-GTGCGGATGAAAGAGTGAAGAAC-3′	
Primer 6	5′-GTGATGATGACCGAGTGAAGAAC-3′	

**Table 2:** Primers used in GATA site mutagenesis. The positions of mutations are underlined.

Position of Mutation	Primer Sequence
−12	Forward: 5′-AAAAAGTGCACCCAGACTTAAGGTTCGTTCTCAATGCTCTGCCG-3′ Reverse: 5′-CGGCAGAGCATTGAGAACGAACCTTAAGTCTGGGTGCACTTTTT-3′
−243	Forward: 5′-GCAGACAAGACAGTTACCACTGGCGCTCTGACGAGAGATTC-3′ Reverse: 5′-GAATCTCTCGTCCAGAGCGCCAGTGGTAACTGTCTTGTCTGC-3′
−449	Forward: 5′-CCTCAGGCCTTACTTCCCAAGAAATCATGTGTCAGTGTTGC-3′ Reverse: 5′-GCAACACTGACACATGATTTCTTGGGAAGTAAGGCCTGAGG-3′

**Table 3:** Sequences of qPCR primers in ChIP analyses.

Promoter	Sequence
*IL5RA* P1	Forward: 5′-CCGTGATGATGAAAGAGTGAAG-3′ Reverse: 5′-GCAGAGCATTGAGAACGAAC-3′
*IL5RA* P2	Forward: 5′-AGGCAAAATACCAAAATGGGC-3′ Reverse: 5′-GCAATGTGCGGTGAAACCTA-3′
*ACTB*	Forward: 5′-TGCCTAGGTCACCCACTAACG-3′ Reverse: 5′-GTGGCCCGTGATGAAGGCTA-3′

## Data Availability

The data presented in this study are available on request from the corresponding author.

## Supplementary Materials

The following are available online at https://www.mdpi.com/article/10.3390/ijms221910245/s1.
