# Supplementary material for: Transcriptional Regulation of the Human IL5RA Gene through Alternative Promoter Usage during Eosinophil Development
Source: Int J Mol Sci. 2021 Sep 23;22(19):10245. doi: 10.3390/ijms221910245 (PMC8549700; doi:10.3390/ijms221910245)
Supplement: Supplementary file 1 [file ijms-22-10245-s001.zip › ijms-1371060-supplementary.pdf]

```
                                    Exon 1                                      Exon 2
P1 mRNA UTR   1 GTTCTCAATGCCCTGCCGCTGCTTCTCATCGCATGGCCACCGCATTTCTCAGGCCAGGCACATTGAGCATTGGTCCTGTGCC 82
P2 mRNA UTR   1                                                    GGCCAGGCACATTGAGCATTGGTCCTGTGCC 31
                                                                   *******************************
```

Figure S1. Sequence alignment of 5' UTRs of *IL5RA* P1 and P2 derived transcripts. Transcript derived from the P1 promoter contains exon 1 which is absent from the transcript derived from the P2 promoter. The sequence of P1 mRNA 5' UTR (NCBI Reference Sequence NM_000564) is shown starting at the transcription start site defined by Sun et al [28] and through part of exon 2 to overlap with sequences published by Zhang et. al [29]. The sequence for P2 mRNA 5' UTR is obtained from Genbank AF017653 [29]. Identical bases between the two transcripts are indicated by the asterisks.
